# Music Listening in Times of COVID-19 Outbreak: A Brazilian Study

**DOI:** 10.3389/fpsyg.2021.647473

**Published:** 2021-05-21

**Authors:** Fabiana Silva Ribeiro, João Paulo Araújo Lessa, Guilherme Delmolin, Flávia H. Santos

**Affiliations:** ^1^Department of Social Science, University of Luxembourg, Esch-Sur-Alzette, Luxembourg; ^2^Graduate Program of Psychology, Universidade São Francisco, Campinas, Brazil; ^3^Department of Psychology, Universidade Municipal de São Caetano do Sul, São Paulo, Brazil; ^4^UCD Music and Math Cognition Lab, School of Psychology, University College Dublin, Dublin, Ireland

**Keywords:** COVID-19, social distancing, depression, music listening, Brazil

## Abstract

The COVID-19 outbreak required diverse strategies, such as social distancing and self-isolation, to avoid a healthcare system crisis. However, these measures have been associated with the onset or increase of anxiety and depression symptoms in the population. Music listening was previously shown to regulate emotion, consequently reducing depression symptoms. Since previous studies with Brazilian samples have already shown a high prevalence of depressive symptoms during the first confinement period, the aim of this study was threefold: (i) to compare groups with severe depression symptoms and no depression in what concerns to demographic and socio-economic factors as well as symptoms of anxiety and resilience levels, (ii) to explore changes in music listening daily routine during the confinement measures by both groups (no depression and severe depression), and (iii) to investigate which were the main factors influencing both two groups to music listening during the COVID-19 pandemic. This cross-sectional study included 494 Brazilian respondents aged 18 years and above. Our online survey comprised demographics, socio-economic, and COVID-19 related questionnaires, with questions regarding music listening used during social distancing measures on which the participants rated how much each of the 41 potential reasons for listening to music changed in importance compared to the situation before the pandemic and also the evaluation of anxiety, depression, and resilience levels. The respondents with severe depression were younger and showed higher levels of anxiety symptoms and lower resilience level. Furthermore, they were increasingly likely to listen to music to feel emotionally better with the situation, to feel comfort, to forget problems, to be energetic, to decrease sad feelings, to relax, to cheer up, to forget concerns, to express feelings, to reduce anxiety, to remember better times, to relieve boredom, to mentally stimulate themselves, and to ward off stressful thoughts compared to the participants with no depression. The exploratory factor analysis (FA) identified four types of music listening functions during social distancing measures: negative mood management, cognitive functioning, positive mood management, and physical involvement, in which the participants with severe depression revealed significant differences compared to non-depressed participants for the negative mood management factor, which shows the importance of music listening to regulate their negative emotions. As a conclusion, we can argue that most of our respondents used music listening to cope with and regulate their moods during confinement, especially those who presented with severe depression symptoms.

## Introduction

The COVID-19 disease was identified in December 2019 in China (Zhu et al., 2020). However, it rapidly spread to other countries, such as Brazil, in which the first case was confirmed on February 25 (Ribeiro and Leist, 2020). On March 11, the World Health Organization declared COVID-19 a pandemic (World Health Organization (WHO), 2020). Since then, strategies to prevent the spread of the virus and the burden on healthcare systems have been implemented globally, such as social distancing, confinement, school closure as well as recommendations to work from home. Nevertheless, several studies have shown a negative impact of the strategies mentioned above on psychological disorders’ burden (Brooks et al., 2020; Campos et al., 2020b). In the following paragraphs, we will introduce the impact of COVID-19 on mental health burden and music listening to regulate emotions.

### COVID-19 Outbreak and Mental Health

Some of the psychological impacts of a pandemic might include the fear of getting sick or dying (Hall et al., 2008), the loss of a loved one (Hawryluck et al., 2005), the loss of income (Van Bortel et al., 2016) as well as decreasing satisfaction in life (Ammar et al., 2020) and well-being (Chtourou et al., 2020; Trabelsi et al., 2021) associated with the experience of social isolation. The lack of reliable and up-to-date information on the recovery cases and possible treatments can increase anxiety, stress (Wang et al., 2020), and depression symptoms (Hyland et al., 2020; Feter et al., 2021). Levels of uncertainty can be aggravated by inaccurate or misleading information about the outbreak in social media (Tang et al., 2018), a factor that has been recurrent in Brazil (Ribeiro and Leist, 2020).

It is necessary to point out that behavioral and mental disorders (BMD) are frequent among Brazilians of working productive age, hindering personal, social, and professional development (Santos and Siqueira, 2010). Thus, epidemiological studies indicate that among people aged 25–54, women are more affected by anxiety and mood disorders, being two to three times more susceptible to depression disorders (Santos and Siqueira, 2010; Carneiro and Baptista, 2012). Besides this, high-intensity stressors can potentially trigger suicidal ideas and suicide attempts, especially among young women with low socio-economic status (Rodrigues et al., 2012). A screening using a probabilistic sample of 5,037 Brazilian adults over 18 showed a prevalence (44.8%) of at least one BMD throughout their life—in which severe depression is one of the most frequent (16.9%) (Viana and Andrade, 2012) among all disorders that can be intensified due to confinement to avoid the spread of COVID-19 (Liu et al., 2020).

In a cross-sectional survey in China, during the COVID-19 outbreak, 53.8% of respondents rated the psychological impact of the epidemic as moderate or severe, and the effect was more significant on women. The results also showed a greater psychological impact of the outbreak and higher stress levels, anxiety, and depression in students (Wang et al., 2020) but lower when compared to developing countries such as Bangladesh (Islam et al., 2020) or even Brazil (Goularte et al., 2021; Ribeiro et al., 2021b).

Moreover, Ribeiro et al. (2021b) explored the impact of COVID-19 restrictions on mental health, especially depression and anxiety symptoms, in 936 Brazilian adults. They showed the prevalence of severe depression symptoms in 66.13% of the population, especially in women and young adults (18–39 years old). Congruently, Goularte et al. (2021), with a larger sample (*N* = 1996), identified that anxiety (81.9%) and depression (68%) were the most common psychological disorders found during the COVID-19 pandemic in Brazilian adults. Furthermore, the pandemic can worsen the condition of patients with pre-existing mental health diseases (Ho et al., 2020; Feter et al., 2021). Given this scenario, studies have demonstrated the effectiveness of Fullana et al. (2020) and recommended activities to prevent or mitigate the impacts of confinement on mental health, such as music listening (Kar et al., 2020).

### Music Listening and Mental Health During the COVID-19 Outbreak

Music is a crucial part of our daily life, and its influence on individuals’ mental health has been consistently shown by previous studies (DeNora, 1999; Sloboda and O’Neill, 2001; Silk et al., 2003; Pan et al., 2020). Saarikallio (2012) proposed that music listening’s effect on emotions has an essential impact on the listener’s mental health, in which music can evoke esthetic pleasure, emotional awareness, and autobiographical memories. Furthermore, she suggests three psychological aspects related to music listening which were organized by observing the results of previous studies. The first is the use of music to express, experience, and/or regulate emotions (Juslin and Västfjäll, 2008; Saarikallio et al., 2020); secondly, music is used as a means for reflection, personal development, relaxation, and mental work; and thirdly, music is used as an element to enhance social bonding, social cohesion, and/or identity.

Studies exploring why subjects listen to music revealed various reasons; for example, in the study of Lonsdale and North (2011), they noticed six motives for music listening, namely: negative mood management (music is utilized to relieve negative feelings and to improve mood), personal identity (music is used for the development of identity or to express a social image for others), surveillance (where music is used to keep up with current events or learn about things), positive mood management (music as a means to reach and enhance a positive mood), interpersonal relationships (music is used to encourage and sustain social contact), and diversion (music as a means to distraction, alleviate boredom, or pass the time). In a study carried out with adults with and without depression, Wilhelm et al. (2013) found 10 main reasons for listening to music as follows: energy and motivation, mood enhancement, relax or reduce stress, inspiration and stimulation, to express, experience, or understand emotion, focus and concentration, to match or reflect the mood, escape, distraction, or immersion, to reminisce, and solace, although all participants reported similar reasons for listening to music. The uses to express, experience, or understand emotion were more prevalent among participants with depression, while people without depression were more likely to endorse the themes of energy, motivation, inspiration, and stimulation.

Nevertheless, Schäfer et al. (2013) argue in their review that surveys diverge in the number of musical functions as well as its dimensions because they are quite analogous concerning social or emotional functions as well as cognitive or self-related purposes and arousal-associated or physiological functions. Notwithstanding, more recently, Sakka and Juslin (2018) evidenced that the most used strategy during music listening was discharging (the release of sad emotions).

Another point is that studies have shown that, in collectivist countries, i.e., Brazil, which prioritizes the group’s aims more than an individual’s needs, the population uses music to reach relaxation, motivation, and happy states, in contrast with individualist countries where people look for sadness–melancholy (Juslin et al., 2016; Saarikallio et al., 2020).

During the confinement and the measures imposed by the COVID-19 outbreak, a sharp increase in new users of musical streaming apps was observed (Hall, 2020); the time dedicated to music listening has also increased (Cabedo-Mas et al., 2020; Krause et al., 2020: Ribeiro et al., 2021a). One possibility is that music listening has been used more frequently to improve affective states. In fact, music listening has been established as one of the means to alleviate the impact of social distancing measures during the COVID-19 pandemic since it reduces loneliness and is a means of distraction (Rodero, 2020), thus increasing mental wellness (Puyat et al., 2020).

Previous systematic reviews demonstrated that music listening can significantly decrease cortisol levels, consequently reducing physiological arousal and stress (Finn and Fancourt, 2018; de Witte et al., 2019). Other literature review showed, through 19 randomized controlled trials, that music listening could decrease subjective anxiety symptoms (Panteleeva et al., 2017). Besides these, music listening is often used by subjects presenting severe symptoms of depression, mainly for mood regulation, relaxation, and reflection (Maratos et al., 2008; Chan et al., 2011; Leubner and Hinterberger, 2017).

A recent study carried out by Pan et al. (2020) evidenced an enhancement in the symptoms of depression by those involved in a program that contrasted daily music listening to controls. The authors’ main rationale was that music is a vehicle of emotion, facilitating the non-verbal expression of emotion and affecting individuals’ innermost feelings without being threatening. For instance, music listening could regulate the mood of participants with depressive symptoms, serving as a tool for emotional catharsis (Pan et al., 2020).

### Study Aims

Although we understand the essential role that music plays under the capacity of emotional regulation, music uses in the daily lives of people with depression in a pandemic context are underexplored.

For this reason, the main objective of this study was to explore changes in music listening during the period of social estrangement due to the COVID-19 pandemic and investigate the main factors for listening to music by participants with severe depression symptoms compared to subjects with no depression symptoms. The scenario of social isolation imposed by the pandemic of the new coronavirus asks questions about the potential effects of listening to daily music, which is relevant to be better understood in a population in which depressive symptoms seem to be highly prevalent (Campos et al., 2020b; Feter et al., 2021). Especially in developing countries like Brazil, the difficulties in offering mental health care to the population are known, so it is crucial to understand the effects of ecologically valid coping strategies, such as listening to music (de Souza and Machado-de-Sousa, 2017; Rzewuska et al., 2017; Fullana et al., 2020).

Our research questions were as follows: (i) are there socio-demographic, anxiety, and resilience level differences between groups (no depression and severe depression)? (ii) are there changes in music listening daily routine during the confinement measures by both groups?; and (iii) which are the main factors underlying music listening by the two groups?

## Methods

### Ethics Statement

The Ethics Committee of São Paulo State University “Júlio de Mesquita Filho” Bauru Campus approved this study (Process: 4.021.098). We obtained electronic informed consent from all participants before they began to fill the online survey.

### Study Design

This quantitative and cross-sectional study was performed to assess the music used by Brazilians during the COVID-19 outbreak. Data were collected using a snowball sampling approach through online-administered questionnaires disseminated in various personal social media platforms by the three authors (FR, FS, and GD). As our sample was composed of volunteers and the questionnaire was available online for a short period of time, it was not possible to establish the sample’s randomness. The inclusion criteria were subjects ≥18 years and in social distancing at the time of study data collection.

### Participants

After excluding repetition (*n* = 23), those that did not accept to participate (*n* = 3), incomplete surveys (*n* = 25), and participants less than 18 years old (*n* = 3), a total of 494 participants who completed at least 90% of the survey were included in the analysis. From the total cohort of participants, 315 were female (63.77%) aged 18–72 years (*M* = 38.84, SD = 14.20) and 179 were male (36.23%) aged 18–76 years (*M* = 38.37, SD = 13.67). Four hundred seventy-seven participants were living in Brazil, while seven were living in North America, one in Asia, and 10 in Europe. We detected that 63.97% (64.76% of females) of our sample presented severe reported depression symptoms.

### Procedure

The data was collected between May 12 and June 12, 2020 through an anonymous online questionnaire available in free software (Google Forms^®^) that was circulated *via* social media (e.g., Facebook, Twitter, Instagram, and other message apps). The study aims, confidentiality, and anonymity were clearly explained before the participants began the online survey. We obtained electronic informed consent from all participants before they began the online survey, and they participated voluntarily in the study. The participants could conclude the survey at any time, and no compensation was given for their participation.

## Materials

### Socio-Demographic and COVID-19-Related Questionnaire

The questionnaire was regarding a follow-up of the rules related to COVID-19, including data regarding schooling, age, marital status, gender, and socio-economic status.

### Music Listening During Social Distancing Measures

These were questions about the use of music listening during social distancing measures, inclusive of 41 questions about their engagement with music (all items are displayed in Supplementary Material 4).

### Mental Health Measures

The Brief Resilience Scale [Smith et al. (2008), Portuguese version from Coelho et al. (2016)]: It assesses respondents’ capacity to deal with social relationships and health situations. It consists of five items, in which the participants respond on a five-point Likert scale ranging from 1 (strongly disagree) to 5 (strongly agree).

General Anxiety Disorder (Spitzer et al., 2006): It assesses generalized anxiety symptoms through seven items. The respondents should reply in a four-point Likert scale going from “not at all” to “several days,” “more than half of the days,” and “nearly every day.” The participants were classified with severe symptoms of anxiety when they presented ≥15 points on the sum of all the items.

Epidemiologic Studies Depression Scale (Radloff, 1977; Filho et al., 2011): This instrument comprises 20 items investigating depressive symptoms in various age groups in epidemiological studies. The participants were classified with severe symptoms of depression when they presented ≥15 points on the sum of all the items.

### Statistical Analysis

Data were analyzed descriptively. When appropriate, comparisons were made between groups with severe and no depression symptoms using *t*-tests for independent samples and chi-square tests (χ^2^). Moreover, to perform the previous quoted analyses, we excluded those respondents who answered: “I don’t know” or “I never had this habit” to be able to observe possible changes after the beginning of the social restriction measures. Descriptive statistics were generated for participants with severe depression and no depression; however, only statistically significant findings are discussed.

Subsequently, factor analysis (FA) was used to investigate the factors related to music listening during the COVID-19 pandemic for the whole sample. To check the best factor structure that would be suitable for the music questionnaire, two different techniques were applied: parallel analysis (PA) and very simple structure (VSS), both on R 3.4 (R Core Team, 2020). PA is a technique based on parameters given through the data used in order to randomly calculate eigenvalues from correlation matrices. On the other hand, VSS compares the fit of different factor solutions and shows these indices so the researcher can choose the best one based on the theoretical expectation. Altogether these two techniques can provide a robust factor solution to be extracted (Campos et al., 2020a; Revelle, 2020).

Then, a FA with the polychoric correlations and maximum likelihood extraction was used from the results of PA and VSS (see the Supplementary Material). After that, factor congruence was done to seek comparisons between the empirical factor loadings for each item and the factor structure’s theoretical factor loadings. In other words, factor congruence correlates the factor loadings found in the FA with each item’s expected factor loadings (i.e., “1” for the theoretical factor, and “0” for the other; Zanon et al., 2018; Revelle, 2020). When the best factor solution was found, Pearson correlation was used to check for external validity evidence, and violin plots were created from the score distribution according to the depression groups’ classification (no depression and severe depression symptoms) for each factor. Finally, the Yuen test for independent groups was used to explore whether differences could exist by comparing the severe depression and no-depression groups for each factor found. All analyses were performed on R 3.4 (R Development Team, 2020) through the packages dplyr (Wickham et al., 2018), ggplot2 (Wickham, 2016), and psych (Revelle, 2020).

## Results

### Socio-Demographics, Anxiety, and Resilience Levels

Table 1 displays comparisons between respondents with severe depression and no depression symptoms during confinement measures. The Student’s *t*-test for independent measurements showed that the members of the severe depression group were younger than those of the no-depression group. Moreover, we detected that participants with severe depression presented lower levels of resilience but higher levels of anxiety symptoms than the group with no depression symptoms. Other significant differences detected by chi-square tests (χ^2^) were severe depression symptoms in groups with less formal education, less income, and whose members were likely to be single. Finally, the subjects with severe depression reported having a harder time staying at home during the social distancing measures than those without depression.

**TABLE 1:** The demographic characteristics of our sample.

**Variables**	**No depression (*n* = 178)**	**Severe depression (*n* = 316)**	***t*_(492)_**	***p***
Age	45.70 (14.58)	34.71 (11.98)	9.03	<0.001
Resilience	18.61 (3.52)	14.84 (4.17)	10.68	<0.001
CES—total	10.08 (3.43)	26.45 (8.85)	29.20	<0.001
GAD-7—total	3.07 (2.63)	8.92 (4.41)	16.16	<0.001

Education	*n(%)*	*n(%)*	χ^2^	

Secondary education	23 (12.92)	96 (30.38)	20.01	<0.001
Undergraduate	59 (33.15)	59 (30.06)		
Post-graduation	96 (53.93)	125 (39.56)		
No wages due to COVID				
No	134 (77.46)	236 (75.64)	0.20	0.65
Yes	39 (22.54)	76 (24.36)		
Familiar income				
1–3 wages	35 (20.35)	98 (31.31)	19.90	<0.001
3–6 wages	51 (29.65)	96 (30.67)		
6–10 wages	33 (19.19)	73 (23.32)		
>10 wages	53 (30.81)	46 (14.70)		
Civil status				
Single	57 (32.02)	174 (55.06)	30.58	<0.001
Married	109 (61.24)	118 (37.34)		
Divorced	9 (5.06)	23 (7.28)		
Widow	3 (1.69)	1 (0.32)		
Stay at home during social distancing has become				
Easier	36 (20.22)	52 (16.46)		
No changes	111 (62.36)	115 (36.39)	45.32	<0.001
Harder	31 (17.42)	149 (47.15)		

### Music Listening During the Pandemic

The second set of analysis using chi-square tests (χ^2^) showed that those participants with severe depression symptoms were more likely to report relying on music listening to change their mood (93.65%) compared to those with no depression (80%) during the confinement, χ^2^(2) = 20.61, *p* < 0.001. Moreover, participants with severe symptoms of depression reported that music was present in their daily life (83%); they were more likely to study or work listening to music (58%) as well compared to subjects without depression (74 and 49%, respectively, χ^2^ = 14.69, *p* < 0.001, χ^2^ = 11.27, *p* = 0.004).

Furthermore, the analysis carried out with the logistic regression detected that only listening to music to change the mood was associated with lower symptoms of depression (*p* = 0.004; OR = 0.36.1; 95% CI = 0.17–0.73).

The chi-square tests (χ^2^), including the responses for the use of music during social distancing measures comparing participants with severe symptoms of depression with those without depression, showed that the former was more likely to report listening to music as much more important to making them energetic, to decrease sad feelings, to take the tension off and relax, to cheer them up, to stop forgetting their concerns, to ward off stressful thoughts, to express feelings, to reduce anxiety, to entertain, to increase self-esteem, to help them think about their lives in a different perspective, to remind them of better times, to relieve boredom, to mentally stimulate, and to surf the Internet. It is a source of inspiration (*p* < 0.004; see Supplementary Table 3).

### Factors Underlying Musical Listening

**FIGURE 1:**
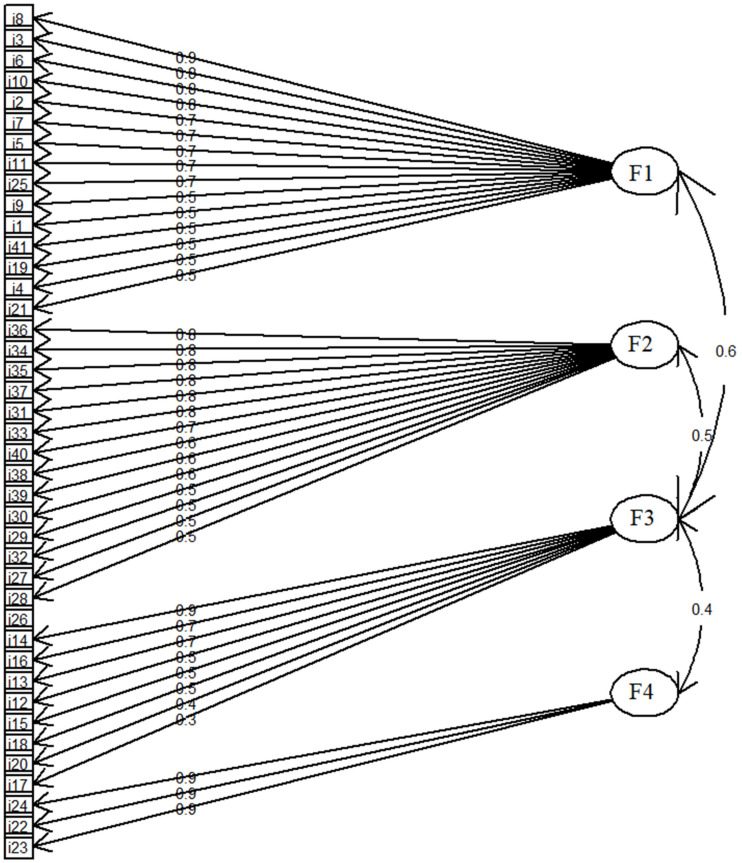
Factor loadings for music listening during pandemics. Fl, negative mood management; F2, cognitive function; F3, positive mood management; F4, physical involvement. All the questionnaire items are displayed in Supplementary Table 4.

The FA conducted on the participants’ responses to the questionnaire items about the use of music during social distancing measures showed four different factors with higher eigenvalues. These together accounted for 46% of the variance present in the participants’ ratings. Factor loadings greater than 0.30 are shown in Figure 1 (for detailed results, see Supplementary Table 4). Specifically, factor 1 might be translated as “negative mood management” since it includes items regarding relieving negative feelings and mood improvement. Factor 2 might be interpreted as “cognitive function” where music is applied to perform cognitive skills, such as reading, arithmetic, memorizing, and learning new content. Factor 3 can be understood as “positive mood management” in which music is used to convey a positive mood, and, finally, factor 4 can be interpreted as “physical involvement.”

As shown in Table 2, the Yuen test for independent groups was used to explore whether differences could exist by comparing the severe depression and no-depression groups for each factor found. We found that the groups differed only in the first factor (negative mood management).

**TABLE 2:** Yuen’s *t*-test for groups for the four factors.

	***T***	**df**	***p***	**Mean difference**	**ξ**
Negative mood management	5.911	256	<0.001	0.420	0.41
Positive mood management	1.526	241	0.13	0.18	0.10
Cognitive function	0.250	198	0.80	0.04	0.026
Physical involvement	0.612	271	0.54	−0.12	0.041

*ξ is the effect-size index for Yuen’s *t*-test.*

**FIGURE 2:**
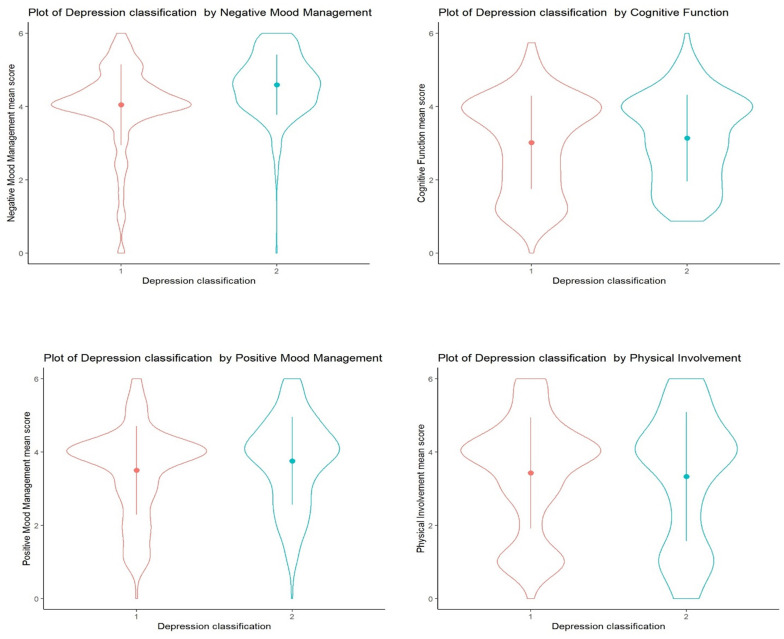
Violin plots representing the distribution of the data for each factor by groups. 1, no depression; 2, severe depression.

In the violin plot (see Figure 2), it is possible to find out the representation of distributions for the responses of each factor by groups and observe that the severe depression group had higher punctuation in what concerns negative mood management compared to the no-depression group, which means that, possibly, the first relies more on music to regulate the member’s negative mood.

## Discussion

Our aims with this study were to explore changes in music listening in individuals experiencing severe levels of depression and those with no depression symptoms and to investigate which were the factors underlying music listening during social distancing measures due to the COVID-19 outbreak by testing if music listening would be diverse from those without depression. In the following sections, we aimed to discuss the results for our three questions.

### Are There Socio-Demographic, Anxiety Symptoms, and Resilience-Level Differences Between Groups (No Depression and Severe Depression) During the COVID-19 Outbreak?

First of all, as we were expecting it, a prevalence of 63.96% for severe depression symptoms was detected, which is higher compared to that of other countries, such as the United States (Fitzpatrick et al., 2020), Ireland (Hyland et al., 2020), Germany (Bäuerle et al., 2020), Spain, and Greece (Papandreou et al., 2020), but in congruence with developing countries such as Bangladesh (Islam et al., 2020) as well as a study carried out in Brazil at the beginning of the pandemic (Campos et al., 2020b). These results might be related to the fear of losing their jobs and/or their beloved ones (Hawryluck et al., 2005), financial insecurity, and fewer resources from governmental measures (Van Bortel et al., 2016). In line with these arguments, we found that those respondents with severe symptoms of depression were more likely to have lower education, have a lower income, and be single. Some possible explanations are the difficulties that the population with lower socio-economic status have to deal with, such as difficulty in accessing the health system, inability to perform confinement for work reasons, and fear of losing their job, making them a more vulnerable group (Campos et al., 2020b).

Furthermore, in Brazil, it was possible to observe discrepant speeches from the power figures of the states and presidency and a lack of up-to-date information regarding the COVID-19 cases, which are features that might also hamper the sense of security and, consequently, the mental health of the respondents (Tang et al., 2018; Wang et al., 2020).

Moreover, we detected that those respondents presenting severe depression during the social distancing measures were younger, with lower resilience and with higher scores on the anxiety scores. These outcomes indicate a difficulty for younger people to cope with stressors and keep psychological distress at lower levels during this pandemic (Chen and Bonanno, 2020), possibly due to uncertainty about their future prospects (Ribeiro et al., 2021b).

### Are There Changes in Musical Listening Daily Routine During the Confinement?

In general, 87% of the participants revealed more engagement with music listening during social distancing measures, which agrees with studies showing increases in the use of music listening during the COVID-19 outbreak (Cabedo-Mas et al., 2020; Krause et al., 2020; Ribeiro et al., 2021a). However, we observed that those participants with severe depression symptoms differed significantly from those without depression regarding music uses during the distancing measures for coping with distressful emotions. For instance, it was possible to see more use in regards the relief of a negative mood (i.e., “to ward off stressful thoughts,” “take the tension off and relax,” “to cheer me up,” “to reduce my anxiety,” “decreases sad feelings,” “stop forgetting my concerns”), which was corroborated by the factor analyses. One possible explanation for differences just in these items might be, as previously shown, that individuals with depression use music to maintain their mood in order to feel validated and to feel acknowledged (Stewart et al., 2019).

Moreover, it is essential to mention that the subject’s music may include benefits in what consists of evoking positive moments, serving as a protective factor against symptoms of depression and improving well-being (Rutter, 2012). Besides these, even the choice of songs that have a negative-mood-matching component can promote a reduction in the intensity of the sad emotions, causing an improvement in depressive symptoms (Davis et al., 2008) due to its esthetically pleasant chords (Sachs et al., 2015). It can also provide moments of distraction beyond what is experienced at the moment by reducing the time spent on rumination (Polanco-Roman et al., 2015).

### Which Are the Main Factors Underlying Musical Listening?

Through the FA, we observed four factors which correspond to “negative mood management,” cognitive enhancing, “positive mood management,” and “physical involvement,” which is in line with previous studies showing the importance of music listening to positive and negative mood management (Lonsdale and North, 2011; Schäfer et al., 2013). Besides this, this study found that the music listening main difference is in managing emotions, specifically for those positive and negative ones, which was also shown by Lonsdale and North (2011). This is important since we can detect those participants with depressive symptoms that could use diverse strategies in dealing with music compared to the non-depressive ones, more specifically to alleviate a negative mood (i.e., depressive symptoms, loneliness, and others) or/and produce or boost a positive mood (i.e., energize, relax, and others). In this context, we detected that individuals with severe depression symptoms respond differently to one of the factors, specifically the “negative mood management,” i.e., included questions related to decreasing of a sad feeling, boredom, stressful thoughts, and anxiety, in comparison to non-depressed participants. This might be explained by the importance of music listening in activating cerebral responses related to rewarding as well as neurophysiological responses such as relaxation (Osuch et al., 2009). Furthermore, people with depressive symptoms would use different strategies, as suggested by Sakka and Juslin (2018), in which the common regulatory strategy was the discharge—in other words, the release of negative emotions, although we could not confirm whether music listening in the participants with severe depression was characterized by adaptive behavior. It is crucial to claim robust findings pointing that personal music choice might improve depressive symptoms (Chan et al., 2011; Pan et al., 2020).

### Strengths and Limitation

As far as we know, this is the first study to establish a model for using music in coping strategies. Therefore, the FA is a strength of this paper since it allowed us to identify the components implicit in the music experience coping strategies during the COVID-19 outbreak. This might guide clinicians and music therapists to use music in helping clients identify their emotions and take control over them (Maratos et al., 2008; Leubner and Hinterberger, 2017; Pan et al., 2020).

Although the number of participants is sufficient for statistical purposes, they provide enough power for the conclusions obtained. A key limitation of the study is the sample size in terms of national representativity. Firstly, it is due to a socio-economic bias, as individuals with lower income usually have less engagement with research—particularly with digital resources; second, despite our advert to participants which said, “the music in the pandemic,” we cannot rule out that the topic attracted more participants with depression than others. Finally, it is essential to highlight that the findings are based on the screening scale of depressive symptoms. It would be desirable to expand the work by testing this model in a clinically diagnosed sample. Moreover, we also need to point out that we did not assess the participants before the beginning of the pandemic. It is possible to suggest that the participants already had mild depressive symptoms, which worsened after the COVID-19 restrictions (Wang et al., 2020). A follow-up would still be necessary to confirm this hypothesis.

## Conclusion

Based on our results, a high percentage of our sample adopted music listening during this context of uncertainty caused by the COVID-19 outbreak to improve their mood and deal with isolation, especially those with severe depressive symptoms.

## Data Availability Statement

The datasets generated for this study are available on request to the corresponding author.

## Ethics Statement

The studies involving human participants were reviewed and approved by Universidade Estadual Paulista/Campus Bauru. The patients/participants provided their written informed consent to participate in this study.

## Author Contributions

FR, FS, and GD designed the study. FS conceived the model for the theoretical framework. FR and JL analyzed the data. FR drafted the initial manuscript. All authors revised the manuscript and approved its final version.

## Conflict of Interest

The authors declare that the research was conducted in the absence of any commercial or financial relationships that could be construed as a potential conflict of interest.
