# Supplementary material for: Music Listening in Times of COVID-19 Outbreak: A Brazilian Study
Source: Front Psychol. 2021 May 21;12:647473. doi: 10.3389/fpsyg.2021.647473 (PMC8177432; doi:10.3389/fpsyg.2021.647473)
Supplement: Supplementary file 1 [file Table_1.pdf]

Supplementary Material

Table S1. Number and percentage of participants for statistically significant items of the questionnaire about listening to music by groups.

Questionnaire Items	Much less important	Less important	Of equal importance	More important	Much more important	$\chi 2$	p
It makes me energetic.							
No depression	21 (14.38)	2 (1.37)	2 (1.37)	91 (62.33)	30 (20.55)	19.28	<.001
Severe depression	18 (7.32)	6 (2.44)	13 (5.28)	120 (48.78)	89 (36.18)		
Decreases sad feelings							
No depression	15 (10.07)	1 (0.67)	2 (1.34)	89 (59.73)	42 (28.19)	15.24	.004
Severe depression	8 (3.57)	6 (2.68)	5 (2.23)	110 (49.11)	95 (42.41)		
Take the tension off and relax.							
No depression	8 (5.48)	2 (1.37)	0	80 (54.79)	56 (38.36)	16.93	.002
Severe depression	3 (1.38)	4 (1.83)	4 (1.83)	87 (39.91)	120 (55.05)		
It is a source of inspiration for me							
No depression	12 (8.05)	0	1 (0.67)	107 (71.81)	29 (19.46)	17.39	.002
Severe depression	13 (5.68)	5 (2.68)	4 (1.75)	125 (54.59)	82 (35.81)		
To cheer me up							
No depression	8 (5.41)	0	0	93 (62.84)	47 (31.76)	18.11	.001
Severe depression	4 (1.83)	3 (1.37)	7 (3.20)	105 (47.95)	100 (45.66)		
Stop forgetting my concerns							
No depression	23 (14.84)	4 (2.54)	1 (0.65)	98 (63.23)	29 (18.71)	28.72	<.001

Severe depression	18 (7.63)	3 (1.27)	8 (3.39)	109 (46.19)	98 (41.53)		
To ward off stressful thoughts							
No depression	14 (9.40)	2 (1.34)	4 (2.68)	92 (61.74)	37 (24.83)	31.52	<.001
Severe depression	10 (4.61)	4 (1.84)	2 (0.92)	85 (39.17)	116 (53.46)		
To express my feelings							
No depression	11 (7.19)	1 (0.65)	1 (0.65)	112 (73.20)	28 (18.30)	17.34	.002
Severe depression	6 (2.64)	4 (1.76)	8 (3.52)	136 (59.91)	73 (32.16)		
To reduce my anxiety							
No depression	16 (10.74)	2 (1.34)	2 (1.34)	88 (59.06)	41 (27.52)	20.14	<.001
Severe depression	12 (5.48)	2 (0.91)	5 (2.28)	91 (41.55)	109 (49.77)		
To entertain me.							
No depression	11 (7.19)	0	1(0.65)	89 (58.17)	52 (33.99)	12.96	.01
Severe depression	2 (0.87)	1 (0.44)	4 (1.75)	132 (57.64)	90 (39.30)		
To increase my self-esteem							
No depression	32 (20.65)	2 (1.29)	6 (3.87)	98 (63.23)	17 (10.97)	11.66	.02
Severe depression	30 (12.30)	3 (1.23)	17 (6.97)	143 (58.61)	51 (20.90)		
Helps me think about my life in a different perspective.							
No depression	21 (13.38)	3 (1.91)	4 (2.55)	101 (64.33)	28 (17.83)	12.18	.01
Severe depression	31 (12.16)	4 (1.57)	12 (4.71)	127 (49.80)	81 (31.76)		
Reminds me of better times							
No depression	20 (12.82)	2 (1.28)	7 (4.49)	90 (57.69)	37 (23.72)	19.87	.001

Severe depression	19 (8.60)	2 (0.90)	8 (3.62)	90 (40.72)	102 (46.15)		
To relieve boredom							
No depression	20 (13.07)	0	3 (1.96)	85 (55.56)	45 (29.41)	21.74	< .001
Severe depression	9 (4.09)	9 (4.09)	5 (2.27)	102 (46.36)	95 (43.18)		
To surf the internet							
No depression	51 (31.29)	2 (1.23)	7 (4.29)	88 (53.99)	15 (9.20)	13.08	.01
Severe depression	55 (19.50)	13 (4.61)	13 (4.61)	156 (55.32)	45 (15.96)		
To mentally stimulate me							
No depression	21 (13.46)	1 (0.64)	2 (1.28)	101 (64.74)	31 (19.87)	14.16	.007
Severe depression	17 (7.02)	2 (0.83)	7 (2.89)	132 (54.55)	84 (34.71)		

The investigation for the factor structure solution happened through two different analysis technique, where the first one is the well-known Parallel analysis (PA) that calculates eigenvalues from randomly generated correlation matrices, and Very Simple Structure (VSS), which compares the fit of the simplified model to the original correlations. PA results showed a solution of up to seven factors and five components. From these results, VSS asked to compare up to 7 factors, which led to a minimum of two factors and a maximum of six factors.

Table S2. shows the fit the data would present according to the number of factors requested.

N	VSS	χ^2	Fit	RMSEA	BIC	SRMR
2	0.93	4322	0.93	0.098	-270	0.066
3	0.93	3082	0.95	0.082	-1268	0.047
4	0.85	2484	0.96	0.074	-1630	0.039
5	0.77	1931	0.97	0.065	-1953	0.031
6	0.75	1610	0.97	0.059	-2050	0.027

Note: N is the number of factors; VSS is the Very Simple Structure complexity.

Because of that, three different factor analyses were done. The first was asked for a three-factor extraction, whereas the second asked for a four-factor extraction and the last one, a five-factor extraction. With the factor loadings of each item computed, the factor congruence was done to check which factor solution would have a better congruence between the theoretical loadings and empirical loadings. So, the four-factor solution showed better results, with coefficients presenting loadings of .90 or higher. Table S3 presents the results of each factor congruence, and Table S4 presents the loadings of the four-factor structure, as well as consistency indexes alpha, G6 and omega.

Table S3. Factor congruence from the three different factors found

Three-factor solution					
	F1t	F2t	F3t		
F1e	**0.88**	0.11	0.38		
F2e	0.00	**0.94***	0.22		
F3e	0.07	0.03	**0.67**		
Four-factor solution					
	F1t	F2t	F3t	F4t	
F1e	**0.94***	0.13	0.16	0.01	
F2e	0.03	**0.96***	0.11	0.01	
F3e	0.22	0.12	**0.91***	0.00	
F4e	0.11	0.04	0.08	**0.96***	
Five-factor solution					
	F1t	F2t	F3t	F4t	F5t
F1e	**0.96***	0.06	0.15	0.00	0.09
F2e	0.00	**0.92***	0.06	0.01	0.18
F3e	0.21	0.08	**0.91***	0.00	0.12
F4e	0.10	0.07	0.08	**0.96***	0.03

F5e	0.15	0.48	0.16	-0.01	**0.85***

Note: ‘t’ for theoretical factors; ‘e’ for empirical factor loadings; values in bold correspond to the congruence consistency between theoretical factor and empirical loadings; * to sign values ≥ .90

Table S4. Factor loadings and consistency indexes for the four-factor solution

items	λ_1 (negative mood management)	λ_2 (cognitive function)	λ_3 (positive mood management)	λ_4 (physical involvement)
1. To relieve boredom	**0,65**	0,02	0,07	0,09
2. It makes me energetic.	**0,52**	-0,07	0,18	0,19
3. Decreases sad feelings	**0,73**	-0,08	0,10	0,06
4. Take the tension off and relax.	**0,84**	-0,06	-0,02	0,00
5. It is a source of inspiration for me	**0,49**	0,12	0,20	-0,04
6. Background to have a more pleasant environment	**0,70**	0,13	-0,07	0,07
7. To cheer me up	**0,82**	-0,01	-0,08	0,03
8. Stop forgetting my concerns	**0,72**	0,01	0,09	-0,02
9. To ward off stressful thoughts	**0,86**	0,03	-0,03	-0,04
10. To express my feelings	**0,53**	0,05	0,25	0,03
11. To reduce my anxiety	**0,77**	-0,02	0,10	0,04
12. To entertain me.	**0,69**	-0,07	0,05	0,07

13. To mentally stimulate me	**0,50**	0,39	0,03	-0,08
14. Helps time pass more quickly	**0,49**	0,04	0,09	0,24
15. To surf the internet	0,23	**0,29**	0,17	0,09
16. To concentrate	0,22	**0,53**	0,12	-0,03
17. To work.	0,19	**0,48**	0,11	-0,03
18. To learn faster	-0,03	**0,53**	**0,32**	0,09
19. To keep the focus.	0,20	**0,55**	0,13	-0,04
20. To perform text reading tasks	-0,05	**0,77**	0,02	0,06
21. To perform graphic tasks	-0,03	**0,53**	0,06	0,08
22. To review material for exams	-0,18	**0,75**	0,07	0,09
23. To write texts.	-0,06	**0,79**	0,00	-0,01
24. To memorize the subject	-0,09	**0,78**	0,03	0,07
25. To read.	-0,04	**0,81**	-0,01	-0,02
26. To do course work.	0,04	**0,77**	-0,09	0,06
27. To develop ideas.	0,33	**0,63**	-0,04	-0,13
28. To think or learn something new.	0,30	**0,59**	-0,01	-0,02
29. Perform numerical tasks	-0,07	**0,71**	0,02	0,06
30. To have company.	0,25	0,01	**0,54**	-0,03
31. To increase my self-esteem	0,15	-0,03	**0,72**	0,06
32. To feel more competent	-0,08	0,05	**0,89**	0,02
33. To understand my feelings	0,34	0,09	**0,45**	0,01
34. To give me the feeling of control	-0,01	0,07	**0,74**	0,04

35. It helps me sleep.	0,12	0,23	**0,32**	0,10
36. Helps me think about my life in a different perspective.	0,35	0,02	**0,45**	-0,03
37. Reminds me of better times	0,50	-0,03	**0,27**	-0,01
38. Helps me keep up the routine	0,24	0,09	**0,39**	0,11
39. Helps me exercise longer	0,02	0,02	-0,03	**0,91**
40. Improves my level of physical endurance	-0,03	0,05	0,08	**0,85**
41. It gives me a good rhythm for physical exercise.	0,05	-0,02	-0,04	**0,93**
α	0.93	0.94	0.93	0.89
G6	0.94	0.95	0.92	0.91
ω	0.95	0.92	0.94	0.86

Note: values in bold to indicate factor loadings ≥ .30

Table S5. Percentage responses for each item of the musical questionnaire.

**Questionnaire Items**	**It was never important**	**Much less important**	**Less important**	**Of equal importance**	**More important**	**Much more important**	**Did not know**
It makes me energetic.	3.29	8.02	1.65	3.09	43.42	24.49	16.05
Decreases sad feelings	3.27	4.70	1.43	1.43	40.70	28.02	20.45
Take the tension off and relax.	1.03	2.27	1.24	0.83	34.50	36.36	23.76
It is a source of inspiration for me	2.68	5.15	1.03	1.03	47.84	22.89	19.38

Background to have a more pleasant environment	1.03	4.12	0.82	1.03	47.12	25.10	20.78
To cheer me up	0.62	2.47	0.62	1.44	40.74	30.25	23.87
Stop forgetting my concerns	2.06	8.45	1.44	1.86	42.68	26.19	19.32
To ward off stressful thoughts	0.62	4.95	1.24	1.24	36.49	31.55	23.92
To express my feelings	1.24	3.52	1.04	1.86	51.35	20.91	20.08
To reduce my anxiety	1.03	5.77	0.82	1.44	36.91	30.93	23.09
To entertain me.	0.62	2.69	0.21	1.04	45.76	29.40	20.29
To have company.	3.12	14.76	2.29	4.37	43.87	18.30	13.31
To increase my self-esteem	3.71	12.78	1.03	4.74	49.69	14.02	14.02
To feel more competent	4.78	19.75	1.66	5.20	45.95	14.14	8.52
To understand my feelings	2.46	9.65	1.64	2.46	52.57	17.25	13.96
To give me the feeling of control	5.99	23.35	1.86	3.31	46.69	12.60	6.20
It helps me sleep.	2.91	27.44	2.08	6.65	42.00	10.81	8.11
Helps me think about my life in a different perspective.	3.10	10.74	1.45	3.31	47.11	22.52	11.78

Reminds me of better times	1.03	8.06	0.83	3.10	37.19	28.72	21.07
Helps me keep up the routine	2.71	14.79	1.67	3.75	45.42	22.08	9.58
Helps time pass more quickly	2.07	11.18	0.41	3.31	42.44	25.67	14.91
Helps me exercise longer	5.19	20.12	3.53	6.64	37.14	14.52	12.86
Improves my level of physical endurance	7.97	23.06	2.73	4.82	40.04	13	8.39
It gives me a good rhythm for physical exercise.	5.88	18.70	2.52	6.09	41.60	14.71	10.50
To relieve boredom	0.42	6.04	1.88	1.67	38.96	29.17	21.88
To surf the internet	1.05	22.22	3.14	4.19	51.15	12.58	5.66
To concentrate	0.62	19.96	1.46	4.37	50.73	14.97	7.90
To work.	1.47	18.03	0.63	3.98	52.83	15.51	7.55
To learn faster	2.93	29.29	1.67	5.44	47.28	9.62	3.77
To keep the focus.	0.84	22.57	1.05	4.64	47.89	17.30	5.70
To perform text reading tasks	2.51	36.40	3.56	6.28	40.59	8.58	2.09
To perform graphic tasks	6.12	31.01	2.32	4.85	41.14	10.76	3.80
To review material for exams	6.78	41.10	2.54	8.05	34.11	5.30	2.12
To write texts.	2.95	37.34	1.69	5.06	39.03	9.49	4.43

To memorize the subject	4.85	40.51	2.11	6.96	37.55	6.12	1.90
To read.	2.09	42.38	2.51	6.05	37.37	7.10	2.51
To do course work.	4,46	35,03	1,06	4,67	43,10	8,70	2.97
To develop ideas.	1.46	21.46	1.46	3.54	45.21	16.46	10.42
To think or learn something new.	1.67	21.04	1.04	3.54	48.13	16.25	8.33
Perform numerical tasks	3.98	40.25	2.94	5.94	37.11	7.13	3.14
To mentally stimulate me	0.83	7.88	0.62	1.87	48.34	23.86	16.60
